# The acceptability of rat trap use over pesticides for rodent control in two poor urban communities in South Africa

**DOI:** 10.1186/1476-069X-11-32

**Published:** 2012-05-03

**Authors:** Rifqah Roomaney, Rodney Ehrlich, Hanna-Andrea Rother

**Affiliations:** 1 School of Public Health and Family Medicine, University of Cape Town, Anzio Rd., Observatory, 7925, Cape Town, South Africa; 2 Population Health, Health Systems and Innovation, Human Sciences Research Council, Cape Town, South Africa

**Keywords:** Rat traps, Illegal pesticides, Informal settlements, Acceptability, Rodent control

## Abstract

**Background:**

Rodent infestations are a public health problem in poor urban communities. The use of illegal street pesticides to control rodent infestations with resulting poisonings is an additional public health concern receiving limited attention in many developing countries, including South Africa.

**Methods:**

Participants in a household intervention in two poor urban areas of Cape Town, South Africa, received two high quality rat traps. Reported in this article are the results of a follow-up survey conducted six months after distribution to assess community perceived acceptability of using rat traps instead of toxic pesticides (N = 175).

**Results:**

Of the 175 respondents that were followed up, 88% used the traps and only 35% continued using pesticides after the intervention. The analysis identified perceived effectiveness of the traps (prevalence odds ratio 18.00, 95% confidence interval 4.62 to 70.14), being male (prevalence odds ratio 8.86, 95% confidence interval 1.73 to 45.19), and the willingness to buy traps from an informal market (prevalence odds ratio 17.75, 95% confidence interval 4.22 to 74.57) as significantly associated with the acceptance of trap use.

**Conclusions:**

Rat traps, when introduced to poor urban communities, are acceptable as an alternative to toxic pesticides for rodent control. Sustainability of trap use, however, needs to be researched, especially cost and cost-benefit.

## Background

Rodent infestations, associated diseases and control measures are a global public health concern receiving little attention and often left to individuals to manage. Rodent infestations predominantly affect the urban poor because conditions in poor communities promote rodent breeding, such as poor sanitation and drainage, open drains, uncollected solid waste, improper storage of food and overcrowding of homes [1,2]. The epidemiology of rodent-borne diseases links rodents with a number of diseases such as, plague, leptospirosis, Lassa Fever, salmonellosis, rat-bite fever, viral hemorrhagic fevers and murine typhus [3-5]. These diseases are transmitted through rodent bites, contamination of food with rodent urine or by rodents acting as vectors for other organisms such as fleas [2-7].

People commonly rely on pesticides (which includes rodenticides) to manage rodent infestations as they are perceived to be the most effective method of control [8]. In poor communities in South Africa, people frequently use ‘illegal street pesticides’ which often are hazardous pesticides meant predominantly for agricultural use but which are decanted into containers without labelling and sold for domestic use at informal markets [9]. These pesticides are cheap, easily available, and effective as they are toxic, but are not meant for nor registered for domestic pest control [10]. A commonly used street pesticide, aldicarb, is so toxic that a 60 mg sachet could potentially kill six children that weigh under 10 kg [10]. The use of illegal pesticides such as aldicarb for rodent control has been linked to human poisonings in poor settings in South Africa, Brazil, Zimbabwe, the United States and Israel [2,10-17]. Other commonly used street pesticides in South Africa, such as chlorpyrifos and methamidophos, are banned in several countries because of the high number of related poisoning cases [10].

Unprotected exposures to pesticides can result in severe acute effects (such as fatal poisoning) and chronic health effects (such as birth defects, cancers, asthma, reproductive complications and neurological defects) [18-22]. Pesticide health effects studies are well documented from agricultural regions [23-27] and from urban areas [28,29]. The high risk of poisoning by rodenticides has been recognised as a public health concern by the United States Environmental Protection Agency (EPA). In 2011, the EPA issued a ban on the residential use of most toxic rat and mouse poisons because of the rash of accidental exposures to these substances [30]. In South Africa, similar action has not been taken even though there seems to be an increasing number of children treated for poisoning attributed to the intoxication of street pesticides [9,10,19].

Non-toxic rodent control methods are needed in these at risk communities as there is an overuse of pesticides for rodent control [8]. There are, however, few studies which document people’s attitudes towards using rat traps instead of pesticides. Studies in rural areas have indicated that rat traps are an acceptable alternative control method for rodents interfering in agricultural production [8,31]. No similar studies have been conducted for rodent problems in urban areas.

The use of non-toxic alternatives such as rat traps requires a shift in intentions for people to accept that these alternatives are as effective and feasible as pesticides. Several factors have been identified that are useful for understanding trap adoption and acceptability in poor communities, such as whether the traps are seen to be effective, whether they are easy to use, whether there is an additional benefit to using them and the extent of the rodent infestation [32-36].

This study aimed to investigate whether households in poor urban communities would use traps as a rodent control alternative and to identify factors influencing respondents’ intention to switch from pesticides to rat traps. It was thus an acceptability study. Determining which factors influence the use of non-toxic rodent control has the potential to lower the use of pesticides, especially street pesticides, and reduce the risk of child poisoning and other risks.

## Methods

This research formed part of a larger study that investigated the link between illegal street pesticides and child poisonings in two poor urban areas in Cape Town, South Africa [9,10]. The larger study identified the study sites, Philippi and Khayelitsha, as areas where numerous child poisonings had occurred due to street pesticides used for rodent control [9]. Both areas have high rat infestations as a result of poor sanitation, infrequent refuse removal and overcrowded living conditions [9]. Households were the units of sampling rather than individuals. The study was approved by the University of Cape Town’s Human Research Ethics Committee.

## Study design

The study design for this research was a cross-sectional survey. At the end of a baseline study, respondents were given an intervention (rat traps). A follow up survey was conducted six months later to assess the use of the rat traps and whether people intended to use traps and/or pesticides in the future (see Additional File 1). This article presents only the findings from the follow-up survey and compares respondents who said they would use rat traps in the future to those who said they would not.

**Figure 1:**
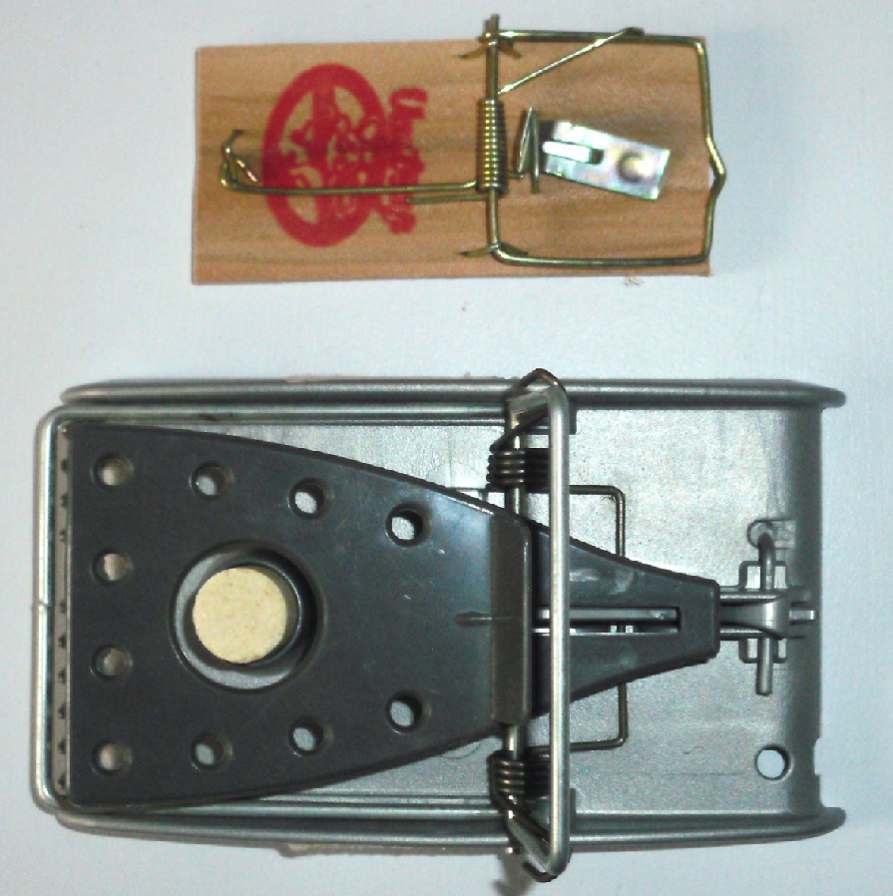
Example of commonly found rat traps (top) and rat traps distributed to respondents (bottom).

A sample of two hundred households was selected, without a formal sample size calculation, as a practical sample size that would yield useful information. Systematic random sampling identified a house from every tenth house starting from the local community centre in each area. The household head or adult at home was interviewed after obtaining written consent*.* After participating in the baseline survey, each family received two free rat traps and instructions on how to set the traps, along with a demonstration from a fieldworker. The rat traps distributed had a higher spring action than conventional traps used in these communities which increased their effectiveness in rat catching (Figure 1). These traps are not usually available in outlets selling conventional rat traps.

Between March and May 2009, 200 face to face interviews were conducted by trained community fieldworkers in Philippi (n = 100) and Khayelitsha (n = 100). The same fieldworkers from the baseline survey were employed six months later (November 2009) to administer the follow-up acceptability survey. The fieldworkers were given addresses to locate the selected houses. From these houses, it was possible to locate 175 of the households that had taken part in the baseline study. The loss to follow-up was due to one questionnaire being misplaced and some families having moved elsewhere. It was not required that the same respondent be followed up, only that they were from the same family that was given the rat trap.

## Analysis

Reported intention to use rat traps in the future was analysed as the main dependent variable and was used as an indicator of acceptability of the rat traps. Reported intention to use pesticides in future was considered as the alternate outcome of interest. The factors related to the reported intended trap use considered were: 1) demographic data (age and sex), 2) whether the respondents reported using the traps, 3) the experienced effectiveness of the traps (whether the traps caught rodents and whether respondents reported any problems using the traps), 4) reported past and present pesticide use, 5) reported perceptions of the price of rat traps (if respondents believed rat traps to be more expensive than pesticides and whether they were willing to buy them) and 6) whether respondents reported still having a rodent problem. Open-ended questions were also asked about why the respondent did not use the rat traps (if such was the case) and what features the respondents liked about the rat traps.

Cross tabulations were used to identify factors related to the reported acceptability of rat trap use after the intervention. A codebook was developed for the qualitative responses and tabulated into themes corresponding to the variables. These were then categorised and converted into categorical data. Data analysis took place in the survey suite of STATA (STATA for Windows, version 10, Stata Corp; College Station, TX).

Multivariate logistic regression analyses were performed to assess the association between the independent and dependent variables. Thus reported “intended trap use” was the main outcome of interest, used to gauge whether respondents found the rat traps to be an acceptable alternative to pesticides. Results considered statistically significant were those with a two-tailed probability of 0.05 or less. To predict use and acceptability of traps, forward stepwise regression was carried out to obtain the predictive model with the best statistical fit which was determined by likelihood ratio tests.

## Results

The median age of the follow-up sample was 31 years (range: 25 to 41 years), with a small female excess (53.8%, n = 93). The median schooling grade attained was Grade 11 (range: Grade 8 to Grade 12). Monthly income levels were low - with the median category being US$129.15 – US$258.18 (range: US$64.57 – US$129.05 to US$258.30 – 387.33US$) (February 2012 exchange rate: US$1.00 = ZAR7.74).

Most of the study respondents reported using the intervention traps (88.0%, n = 154) (Table 1). Most respondents who used the traps reported that they caught rodents (96.1%, n = 148), confirming their effectiveness. The variables “used trap” and “trap caught rodents” were highly collinear and thus “used trap” was left out of further analyses. Most respondents reported they would use rat traps in the future (84.5%), compared to a third who reported they would continue using pesticides (29.2%). Most respondents reported using pesticides before the trap intervention (78.3%), with significantly fewer reporting using pesticides at the time of the follow-up survey (34.5%). More than half of the baseline pesticide users (57.6%) were not using pesticides at follow up. Several respondents (21.7%) reported that they intended to use both pesticides and rat traps in the future (see Additional File 2).

Whether the traps had caught rodents was strongly associated with the reported intention to use traps in the future [prevalence odds ratio (POR) 18.0, 95% confidence interval (CI) 4.6 to 70.1] (Table 2). The reported willingness to buy a rat trap from an informal market where street pesticides are commonly sold (e.g. taxi ranks) was strongly associated with the reported intention to use traps (POR 17.8, 95% CI 4.2 to 74.6). Males were 8.9 times more likely than females (95% CI 1.7 to 45.2) to report the intention to use traps in the future. Pesticide use at the time of follow up was strongly associated with the intention to continue using pesticides in the future (POR 58.2, 95% CI 19.7 to 172.0) (not shown in Table 2).

From the non-prompted responses, of the total sample, 85% (n = 148) of respondents reported that they liked the traps. Specifically, respondents reported that liked the fact that the traps were efficient at catching rodents (33.1%, n = 49), that the traps were safer than pesticides (18.0%, n = 26) and that the rodent carcass was easy to find and remove (18.0%, n = 25). This was in contrast to looking for the carcasses, for example in the roof, as was the case with pesticides. Furthermore, 14% (n = 21) reported that they liked the power and look of the trap, 10% (n = 15) reported that there were fewer rats seen when traps were used and 8% (n = 12) reported that they were easy to use.

Only 18 respondents indicated that they had a problem when using the traps and from the small sample, most of these were female (77.8%, n = 14). Of the 18 respondents who had reported problems with the traps, 50% reported that they were scared to use the traps or scared that their children might get hurt by them and, 22% indicated that they did not know how to use the traps. A further 11% indicated that the trap caught rodents too slowly and two indicated that they had “mechanical” problems (e.g. one person commented that “sometimes the bigger rats will move the traps and you find it somewhere else”). Another respondent noted that “at first everyone was scared of it until our big brother came and did it for us. Now all of us are using it”.

**Table 1:** Follow-up results describing respondents’ intentions and attitudes (N = 175)

**Data**	**Variable**	**Total (n)**	**Yes (%)**
**Dependent variable**	Will you continue using rat traps for rats or mice?	174	84.5
	Will you continue to use pesticides (poison/chemicals) bought at shops or taxi ranks to kill rats or mice?	161	29.2
**Predictor Variables**	Have you used the rat traps since they were given to you?	175	88.0
	Did the traps catch any rats or mice?	174	85.1
	Were you using pesticides to kill rats or mice before you got these rat traps?	175	78.3
	Are you still using pesticides to kill rats or mice?	171	34.5
	Do think it is more expensive to buy rat traps compared to pesticides monthly?	167	62.9
	Are rats and mice still a problem in your house?	171	44.4
	Would you buy a rat trap from a taxi rank/informal market?	173	79.2
	Did you have problems or difficulties using the traps?	174	10.9

**Table 2:** Models describing intentions of rat trap use

**Variable**	**Full Model POR (95% CI)**	**Stepwise Regression Model POR (95% CI)**
Traps caught rodents	**16.02 (3.14-81.62)**	**18.00 (4.62-70.14)**
Current pesticide use	0.77 (0.16-3.57)	-
Traps more expensive	0.86 (0.18-4.03)	-
Current rodent infestation	0.88 (0.16-4.62)	-
Would buy trap from taxi rank	**11.17 (2.34-53.32)**	**17.75 (4.22-74.57)**
Gender (male)	**6.23 (1.04-37.09)**	**8.86 (1.73-45.19)**
Had problems with traps	0.39 (0.05-2.80)	-
Age	0.98 (0.92-1.04)	-

POR = Prevalence odds ratios.

## Discussion

The majority of respondents (78.3%) reported using pesticides at baseline. Although there was a significantly strong association between pesticide use at baseline and follow up, more than half of the people using pesticides at baseline stopped using pesticides at follow up. This demonstrates that many pesticide users were willing to give up their pesticide use and that it was quite rare for non-pesticide users to start using pesticides. Overall, just over a third (34.5%) of participants reported using pesticides at follow up and even fewer (29.2%) reported that they intended to use pesticides in the future. Of those that still intended to use pesticides, the majority intended to also use rat traps. This indicates that even when an individual is convinced of the effectiveness of pesticides, they may still be willing to simultaneously try alternatives.

In the baseline survey, only about a quarter (24.7%, n = 44) of the respondents had ever used rat traps and less than half of them were using rat traps at the time of the baseline survey (n = 19). With the majority of respondents (84.5%) in this acceptability study reporting intention to use traps in the future, the results indicate a general willingness to change from using street pesticides to rat traps for rodent control.

In order for rat traps to be a viable public health intervention to prevent rodent-borne diseases and pesticide poisonings, acceptability factors need to be taken into account. The factors identified as promoting acceptability were: whether the trap caught rodents, the gender of the user and willingness to purchase a trap from the same informal vendor locations where street pesticides are purchased.

Whether the trap caught rodents or not was strongly associated with trap acceptability. Only 4% of respondents who used traps did not catch rodents with their trap. This highlights the high levels of rodent infestations, which explains why so many people use toxic pesticides. The perceived trap effectiveness was influenced by the type of trap used. The commonly used and widely accessible traps in most South African supermarkets and hardware stores are wooden traps (Figure 1) that were described by respondents to be ineffective in killing rodents and tended instead to maim them, particularly the large rats found in South Africa. The study traps were designed to be effective in that they are made of a heavy duty plastic, had a fast and sensitive spring action, and a serrated edge (Figure 1). Thus in order for the intervention to be accepted, the product needs to be of a better quality than that which is currently used or available in the most accessible shops, especially in poor urban areas. In addition, qualitative data suggest that being able to easily locate the rodent carcass was viewed as an advantage. The odour of decaying rodent carcasses in inaccessible places is a noted drawback of pesticide use and could be used as a reason to encourage rat trap use instead of pesticides. Thus the traps have multiple benefits.

Males were more likely to indicate that they would use traps in the future. This may reflect societal roles as males may be looked upon as protectors and expected or see it as their duty to eradicate larger pests. Since traps require some physical strength to set, this may reinforce the perception that it is a male’s responsibility to do so. Of those that had problems, females (78.9%) had more problems using the traps than males (21.1%) some admitting that they were scared to use them as they could hurt them or their children. The use of traps may result in injured fingers; however this is a lesser hazard than pesticide poisoning. The qualitative data did not suggest that any children were harmed by the rat traps. The gender finding suggests that in future interventions, effort should be put into making sure that females are given extra support when shown how to use traps. For example, an informal vendor selling rat traps could demonstrate how to set traps and have a potential customer set it under their supervision, until the potential customer is confident in doing it by him or herself. Women could also be advised on how to use rat traps more safely by putting them in places inaccessible to children or putting the traps out at night only.

The questions asked relating to cost and buying traps were hypothetical as respondents were given the traps for free. The willingness to buy traps at an informal market/local taxi rank (where street pesticides are sold) was strongly associated with rat trap acceptability. Many people indicated that they were willing to buy traps at informal markets. However, when asked what they were willing to pay for traps, many quoted prices that were much lower than the actual cost of the traps but higher than the current prices for street pesticides. Illegal pesticides are cheap (US$0.13- US$0.26) [9]. Traps represent an investment because they are more expensive. However, rat traps can last for several months and can be reused, whereas people need to continually purchase pesticides. The cost-benefit of investing in a trap may thus need to be marketed as a way to promote trap use. Traps could compete with pesticides if the price of traps were subsidized by government, or by the industry whose agricultural pesticides are being used illegally for domestic rodent control. The affordability of traps is an important factor as this may affect the sustainability of its use.

The study had some limitations, particularly regarding the accuracy of information elicited. The data were self-reported and the purpose of the project was known to participants. Their answers could thus have been affected by desirability bias, although fieldworkers were trained to ensure that they did not prompt respondents for answers. Also, the person who was interviewed in the household may not have been responsible for pest control for the family.

## Conclusions

With climate change predicted to increase levels of pest infestations and pesticide use in poor urban communities [37], it is important to promote the use of alternative non-toxic rodent control methods. The sustained use of rat traps in poor communities through an integrated approach to rodent control that involves ministries of health, community members, non-governmental organisations and other relevant stakeholders could assist in decreasing the double health burden caused by exposure to toxic pesticides and rodent-borne diseases. Rodent control with traps needs to occur together with public health measures aimed at alleviating the underlying factors contributing to rodent infestations, for example, through improving sanitation and waste control.

Most important, the study suggests that rat traps are an acceptable alternative as the majority of respondents used the traps to catch rodents while decreasing their reliance on pesticides. These findings could be applicable to other poor communities with rodent infestations and access to cheap street pesticides. There is potential for rat traps to be widely accepted if high quality rat traps could be made available and at the same time the barrier of the high cost of such traps could be overcome.

Studies are needed to determine if sustained trapping in poor urban communities reduces rodent populations and pesticide use over the long-term. As rodent control is an important public health disease prevention measure, there is a need to advocate for the accessibility of rat traps and cost reduction of rat traps in these impoverished urban communities, as well as policies to restrict residential use of hazardous pesticides.

## Abbreviations

CI: Confidence Interval; EPA: Environmental Protection Agency; POR: Prevalence Odds Ratio.

## Competing interests

The authors declare that they have no competing interests

## Authors’ contributions

H-AR was the principal investigator of the project, conceptualised the project and methods used, developed the protocol, secured funding, supervised data collection and analysis of data and was involved in the research, write up and editing. RE gave input on the statistical analysis, writing and editing. RR analysed the data, did the write up of the article and editing. All authors read and approved the final manuscript.

## Supplementary Material

Additional file 1 Follow up questionnaire Click here for file

Additional file 2 **Supplementary data.** Selected tables not presented in main article Click here for file
