# Supplementary material for: The acceptability of rat trap use over pesticides for rodent control in two poor urban communities in South Africa
Source: Environ Health. 2012 May 3;11:32. doi: 10.1186/1476-069X-11-32 (PMC3508837; doi:10.1186/1476-069X-11-32)
Supplement: Additional file 1 — Follow up questionnaire [file 1476-069X-11-32-S1.docx]

**Appendix 1**

1. **Questionnaire**

**Follow-up Use of Rat Traps Questionnaire - 2009**

**Respondent Number:** **Date:**

**Interviewer’s initials:** ___________

**Location: ____________ Address: ____________________________________**

1. **Date of Birth**:______________________________(Age_________)
2. **Gender: Female Male**
3. Went to school? Yes__________ No___________
4. Highest standard/grade passed at school:______________________

5) How many rat traps have you used on your property since May this year?

0___ 1___ 2___ 3___ 4___ 5___ more than 5___

5a) How many were given to you from UCT? _____

5b) Have you bought any rat-traps since May? If yes how many? ________

6) Have you used the UCT rat traps since they were given to you? Yes No

7) Did the traps catch any rats or mice? Yes No

If yes, how many?

|  | Rats caught per week  Picture A | Rats caught per week  Picture B | Mice caught per week  Picture C |
| --- | --- | --- | --- |
| None |  |  |  |
| Seldom |  |  |  |
| One a week |  |  |  |
| 2-3 a week |  |  |  |
| 5-10 a week |  |  |  |
| Too many to count |  |  |  |

8) What were you using to kill rats or mice before you got these rat traps? (tick)

Pesticides____________ Nothing_____________ Other_____________

If you were using a pesticide was it one of these? [can tick more than one]

| Product used from Pink Point Chart | 5 |
| --- | --- |
| 1 | 6 |
| 2 | 7 |
| 3 | 8 |
| 4 | 9 |

Other: __________________________________

9) Are you still using pesticides to kill rats or mice? Yes No

9a) If yes, indicate which pesticides by using the point chart: [can tick more than one]

| Product used from Pink Point Chart | 5 |
| --- | --- |
| 1 | 6 |
| 2 | 7 |
| 3 | 8 |
| 4 | 9 |

10) Which is better at killing rats and mice? ____ pesticides ____ rat traps

*[If in question* ***10****, the respondent says rat trap are better, whereas in* ***6*** *she said she never used the rat traps that were give to her, ask her how she knows that rat traps are better at killing rats and mice]*

*__________________________________________________________________________________________________________________________________________*

11) Please explain why?

____________________________________________________________________

____________________________________________________________________

12) Did you have problems or difficulties using the traps? Yes No

If yes, Explain: _____________________________________________________________

____________________________________________________________________

____________________________________________________________________

13) What did you like about the traps?

__________________________________________________________________________________________________________________________________________

14) What did you not like about the traps?

__________________________________________________________________________________________________________________________________________

15) Will you continue using traps for controlling rats or mice? Yes No

15a) If no, why not?

__________________________________________________________________________________________________________________________________________

16) Will you continue to use pesticides (poison / medicine / chemicals) bought at shops or taxi ranks to kill rats and mice? Explain why or why not?

___________________________________________________________________

___________________________________________________________________

17) How much would you pay for a rat-trap? ________________________________

18) Which is more expensive, rat traps or pesticides? rat-traps_______ pesticides____________

19) Describe how you used your trap (explain step by step and in detail) and then what you do after a rat/mouse has been caught in it.

__________________________________________________________________________________________________________________________________________

20) Are rats and mice still a problem in your house?

__________________________________________________________________________________________________________________________________________

21) Why do you think you have a problem with pests (e.g., rats, cockroaches, flies) in your home?

__________________________________________________________________________________________________________________________________________

22) Would you buy a rat trap from a taxi rank or street market? Yes No

__________________________________________________________________________________________________________________________________________

23) Do you have any other comments or suggestions you would like to make regarding using pesticides and rat traps?

__________________________________________________________________________________________________________________________________________

24) What is your monthly household income?

| **Monthly Household Income** | **Please tick** |
| --- | --- |
| **R0 - 499** |  |
| **R500 - 999** |  |
| **R1000 - 1999** |  |
| **R2000 - 2999** |  |
| **R3000 - 3999** |  |
| **R4000 - 4999** |  |
| **R5000-5999** |  |
| **R6000 or more** |  |

25) How much do you spend a month on pesticides?

| **Monthly Spend on Pesticides** | **How much do you spend per month on all kinds of pesticides for Cocroaches, ants, flies etc.**  **Please Tick** | **Since May how much have you spent /month on pesticides to kill rats and/or mice.**  **Please tick** |
| --- | --- | --- |
| **R0** |  |  |
| **R1-R5** |  |  |
| **R6-R9** |  |  |
| **R10-R29** |  |  |
| **R30-R49** |  |  |
| **R50-R69** |  |  |
| **R70-R99** |  |  |
| **R100 - R149** |  |  |
| **R150 -R199** |  |  |
| **R200 or more** |  |  |

**Thank you**
