# Supplementary material for: The acceptability of rat trap use over pesticides for rodent control in two poor urban communities in South Africa
Source: Environ Health. 2012 May 3;11:32. doi: 10.1186/1476-069X-11-32 (PMC3508837; doi:10.1186/1476-069X-11-32)
Supplement: Additional file 2 — Supplementary data. Selected tables not presented in main article [file 1476-069X-11-32-S2.docx]

**Appendix 2: Selected tables not presented in main article**

**High levels of pesticide use prior to being given rat traps:**

**Table A1 Main method of rodent control described by participants as having been used prior to study**

| **Method of rodent control** | **Frequency** |
| --- | --- |
| Nothing | 36 (21%) |
| Pesticide | 137 (78%) |
| Cats | 2 (1%) |
| **Total** | **175 (100%)** |

**Females reported more problems using the traps:**

**Table A2: Problems described with the rat traps by gender**

|  | **No problems with traps** | **Problems with traps** | **Total** |
| --- | --- | --- | --- |
| **Female** | 78 (84%) | 15 (16%) | 93 (100%) |
| **Male** | 75 (95%) | 4 (5%) | 79 (100%) |
| **Total** | 153 (89%) | 19 (11%) | 172 (100%) |

**Bivariate tables relevant to Table 2 of main article**

**Table A3: Intention to use traps by whether traps caught rodents**

|  | **Did not intend to use traps** | **Intended to use traps** | **Total** |
| --- | --- | --- | --- |
| **Trap did not catch rodents** | 20 (77%) | 6 (23%) | 26 (100%) |
| **Traps caught rodents** | 0 (0%) | 148 (100%) | 148 (100%) |
| **Total** | 20 (11%) | 154 (89%) | 174 (100%) |

**Table A4: Intention to use traps by willingness to buy trap from an informal vendor**

|  | **Did not intend to use traps** | **Intended to use traps** | **Total** |
| --- | --- | --- | --- |
| **Would not buy trap** | 21 (58%) | 15 (42%) | 36 (100%) |
| **Would buy traps** | 6 (4%) | 131 (96%) | 137 (100%) |
| **Total** | 27 (16%) | 146 (84%) | 173 (100%) |

**Table A5: Intention to use traps by gender**

|  | **Did not intend to use traps** | **Intended to use traps** | **Total** |
| --- | --- | --- | --- |
| **Female** | 19 (20%) | 74 (80%) | 93 (100%) |
| **Male** | 7 (9%) | 72 (91%) | 79 (100%) |
| **Total** | 26 (15%) | 146 (85%) | 172 (100%) |

**Table A6: Bivariate tables of intention to use and reported use of pesticides**

|  | **No intention to use pesticides** | **Intention to use pesticides** | **Total** |
| --- | --- | --- | --- |
| **No use of pesticides at follow up** | **97 (95%)** | **5 (5%)** | **102 (100%)** |
| **Use of pesticides at follow up** | **14 (25%)** | **42 (75%)** | **56 (100%)** |
| **Total** | **111 (70%)** | **137 (85%)** | **161 (100%)** |

**Table A7: Intention to use traps by intention to use pesticides (co-intention of use)**

|  | **No intention to use traps** | **Intention to use traps** | **Total** |
| --- | --- | --- | --- |
| **No intention to use pesticides** | 12 (11%) | 102 (89%) | 114 (100%) |
| **Intention to use pesticides** | 12 (26%) | 35 (74%) | 47 (100%) |
| **Total** | 24 (15%) | 137 (85%) | 161 (100%) |

**Table A8: Pesticide use at follow up by pesticide use before the intervention**

|  | **Did not use pesticide at follow up** | **Used pesticide at follow up** | **Total** |
| --- | --- | --- | --- |
| **Did not use pesticide before intervention** | 33 (97%) | 1 (3%) | 34 (100%) |
| **Used pesticide before intervention** | 79 (58%) | 58 (42%) | 137 (100%) |
| **Total** | 112 (65%) | 59 (35%) | 171 (100%) |
